# 

**Published:** 1946

**Authors:** 


### Local Medical Notes

THE thirty-fifth Long Fox Memorial Lecture was delivered in the University of Bristol on Tuesday, November 19th, by Dr. Kenneth Bergin, on "Some Aspects of Aviation Medicine." Professor Perry took the Chair and an audience of twenty-six persons attended.

UNIVERSITY OF BRISTOL.

The following Lecturers have been appointed: *In Anatomy:* E. J. Field, M.B., M.S. *In Physiology:* Anne S. Cole, B.Sc. ; O. A. Trowell, M.A., M.D., F.R.S.E. ; D. N. Walder, M.B., Ch.B. *In Pathology:* F. J. W. Lewis, M.B., Ch.B. ; N. G. B. McLetchie, M.D. *In Surgery:* H. J. Croot, M.B., B.S., F.R.C.S. ; (Special Lecturer) R. H. R. Belsey, M.B., M.S., F.R.C.S. *In Physical Medicine:* G. D. Kersley, M.D., F.R.C.P. *In Ophthalmology:* A. Palin, M.A., B.M., B.Ch., F.R.C.S.E. *In Anæsthetics:* R. Woolmer, B.A., B.M., B.Ch., D.A.

Mr. W. J. Lennox, B.D.S., has been appointed Honorary Consulting Surgeon, Bristol University Dental Hospital.

MEDICAL POSTGRADUATE STUDIES.

The Department of Medical Postgraduate Studies of the University of Bristol was founded in 1919 by the enterprise of Professor Walker Hall. During the war years, postgraduate work was suspended, but with the cessation of hostilities the department was selected to implement the Ministry Scheme for the employment of demobilized officers. The initial organization was undertaken by Professor Bruce Perry, F.R.C.P., who had held the appointment of Director since 1936 and in September, 1946, he was succeeded by Mr. A. J. Wright, F.R.C.S.

The appointments made by the Ministry fall into one of three categories :—

*Class I.*—Doctors recruited within a year of qualification, the appointment to be held for one period of six months.

*Class II.*—Doctors established in general practice, who attend for a two weeks' course or alternatively twenty-two half-day sessions.

*Class III.*—Doctors who on recruitment were being trained as specialists, the appointment to be held for a period of six months which can be extended.

Two hundred and twenty-six appointments have now been made as follows : Class I, 142 ; Class II, 21 ; Class III, 63.

In order to accommodate the large numbers of doctors who were applicants for hospital appointments, the hospitals in many departments doubled their resident staff and in some departments even exceeded this. These latter appointments are known as supernumerary posts. There exist now therefore the established resident appointments of the hospital made by the hospital, but recognized for the Ministry benefits, and the supernumerary appointments made through the postgraduate department.

The University acts for all Hospitals in the South-West region, and to date appointments have been made at Bristol, Bath, Gloucester, Cheltenham, Stroud, Salisbury, Weston-super-Mare, Bridgwater, Taunton, Exeter, Torquay, Plymouth, Truro, and Redruth, the success of these appointments being assured by the excellent co-operation given by the hospital authorities.

As far as vacancies and circumstances permit, appointments are made at those hospitals for which application is made, although in certain instances where there is a waiting list, some delay has been unavoidable.

The returning General Practitioner was first catered for by a two-weeks set course. It was subsequently found that General Practitioners wished to attend a course with the minimum of delay after demobilization, and a standing course was therefore instituted which ran continuously until the summer vacation.

The need for such a course has now ceased and it is planned to hold a set two-weeks' refresher course which will be available to both General Practitioners and those demobilized from the Forces. This course will be divorced from the normal undergraduate teaching and will be designed to cover all branches of medicine which are considered to be of value to the General Practitioner. Clinical demonstrations will be held in the departments of the Bristol Royal and City of Bristol Hospitals and Winsley Sanatorium.

The University has reintroduced the Course for the Diploma in Public Health (D.P.H.), and has instituted a Certificate in Public Health (C.P.H.). It has also instituted Diplomas in Psychological Medicine (D.P.M.), and Medical Radiology (D.M.R.), the qualification being in either Radio-Diagnosis or Radio-Therapy. Courses for all the above commenced this University session together with a course for Part I of the Diploma of Physical Medicine.

In addition to enquiries regarding the various aspects of post-graduate work, enquiries are also received relating to assistantships and partnerships in General Practice.

The Department is now working towards the development of further postgraduate meetings, both of a specialist and general nature.

### BRISTOL ROYAL HOSPITAL.

The following appointments have been made : *Physicians.*—G. E. F. Sutton, M.C., M.D., M.R.C.P. ; H. J. Orr-Ewing, M.C., M.D., F.R.C.P. *Surgeons.*—W. A. Jackman, T.D., F.R.C.S. ; G. M. FitzGibbon, M.D., F.R.C.S. ; R. V. Cooke, Ch.M., F.R.C.S. ; R. G. Paul, F.R.C.S. *Orthopædic Surgeon.*—K. H. Pridie, M.B., Ch.B., B.S., F.R.C.S. *Obstetric Surgeon.*—F. J. Hector, M.D., F.R.C.S.

**Royal College of Obstetrics and Gynæcology.**—Dr. Mabel Potter has been elected F.R.C.O.G.

### OBITUARY

As we go to press we learn with sorrow of the death of Dr. C. E. K. HERAPATH, M.C., M.D. An Obituary notice will appear in our next number.

